# Seasonal dynamics of *Anopheles stephensi* and its implications for mosquito detection and emergent malaria control in the Horn of Africa

**DOI:** 10.1073/pnas.2216142120

**Published:** 2023-02-15

**Authors:** Charles Whittaker, Arran Hamlet, Ellie Sherrard-Smith, Peter Winskill, Gina Cuomo-Dannenburg, Patrick G. T. Walker, Marianne Sinka, Samuel Pironon, Ashwani Kumar, Azra Ghani, Samir Bhatt, Thomas S. Churcher

**Affiliations:** ^a^Medical Research Council Centre for Global Infectious Disease Analysis, School of Public Health, Imperial College London, London W2 1PG, UK; ^b^Department of Biology, University of Oxford, Oxford OX1 3SZ, UK; ^c^Royal Botanic Gardens Kew, Richmond, Surrey TW9 3AQ, UK; ^d^United Nations Environment Program World Conservation Monitoring Centre, Cambridge CB3 0DL, UK; ^e^Vector Control Research Centre, Puducherry 605006, India; ^f^Section of Epidemiology, Department of Public Health, University of Copenhagen, Copenhagen 1353, Denmark

**Keywords:** *Anopheles stephensi*, malaria ecology, urban malaria, population dynamics, epidemiology

## Abstract

Invasion of the malaria vector *Anopheles stephensi* across the Horn of Africa threatens control efforts across the continent, particularly in urban settings where the vector is able to proliferate. Malaria transmission is primarily determined by the abundance of dominant vectors, which often varies seasonally with rainfall. However, it remains unclear how *An. stephensi* abundance changes throughout the year, despite this being a crucial input to surveillance and control activities. We collate longitudinal catch data from across its endemic range to better understand the vector’s seasonal dynamics and explore the implications of this seasonality for malaria surveillance and control across the Horn of Africa. Our analyses reveal pronounced variation in seasonal dynamics, the timing and nature of which are poorly predicted by rainfall patterns. Instead, they are associated with temperature and patterns of land use; frequently differing between rural and urban settings. Our results show that timing entomological surveys to coincide with rainy periods is unlikely to improve the likelihood of detecting *An. stephensi.* Integrating these results into a malaria transmission model, we show that timing indoor residual spraying campaigns to coincide with peak rainfall offers little improvement in reducing disease burden compared to starting in a random month. Our results suggest that unlike other malaria vectors in Africa, rainfall may be a poor guide to predicting the timing of peaks in *An. stephensi*-driven malaria transmission. This highlights the urgent need for longitudinal entomological monitoring of the vector in its new environments given recent invasion and potential spread across the continent.

## Significance

Invasion of the malaria vector *Anopheles stephensi* across sub-Saharan Africa poses a threat to disease control efforts, particularly in cities where malaria transmission has historically been low but where this invasive vector can thrive. We collate longitudinal catch data to systematically characterize the species’ seasonal dynamics in areas at risk of invasion, which is necessary to guide surveillance and control activities. *An. stephensi*’s temporal abundance is highly variable and, in contrast to dominant vectors across Africa, poorly predicted by patterns of rainfall, instead being shaped by temperature and patterns of land use. This variation has material consequences for effective control of this invasive vector and highlights an urgent need for longitudinal entomological monitoring of the vector in its new environments.

There has been an estimated 40% reduction in the burden of malaria since 2000, predominantly due to significant scale-up of control interventions (1). Increasing urbanization of Africa’s human population [31 to 43% between 1990 and 2018, with >60% expected to live in urban areas by 2050 (2)] is also thought to have indirectly contributed to reductions in disease burden. Previous work has found significantly lower entomological inoculation rates (EIRs) in urban compared to rural settings (3, 4). This is thought to be underpinned by factors including differences in housing quality (5, 6), reduced suitability of habitats for *Anopheles* breeding in urban settings (7–9), better access to treatment (10), and higher population densities leading to lower mosquito-to-human ratios (and reduced transmission) (11). While these trends are not always consistently identified [including surveys where prevalence of malaria is higher in urban areas than in surrounding locations, (12, 13) or previous work highlighting that *Anopheles gambiae s.s.* can adapt to breeding in polluted water characteristic of urban environments (8, 14)], increasing urbanicity across Africa is anticipated to complement planned scale-up of malaria control interventions aimed at achieving the targets outlined in the World Health Organization’s 2030 Global Technical Strategy for Malaria (15).

This beneficial impact of increasing urbanization on malaria burden is contingent on urban settings remaining as areas of comparatively low transmission. This is currently under threat in Africa because of the invasion and establishment of *Anopheles stephensi*, a malaria vector that is potentially capable of thriving in urban areas of the continent (16). There are three known forms of the species (“type,” “intermediate,” and “mysorensis”) found across its native range in South Asia. The mysorensis form is predominantly found in rural settings, is highly zoophilic, and typically possesses a low vectorial capacity (17). By contrast, the type and intermediate forms represent efficient vectors capable of transmitting both *Plasmodium falciparum* and *Plasmodium vivax* (18–20) in urban environments*.* This ability to proliferate in urban locations distinguishes this species from other malaria vectors in sub-Saharan Africa and is thought to be underpinned by an increased tolerance for breeding in polluted water sources (21) and the superior ability to utilize the purpose-built water storage tanks present in many urban settings (22, 23). Indeed, while several studies across Africa have identified the potential for vectors already endemic to the continent (especially the *An. gambiae* complex) to adapt to urban aquatic habitats and drive malaria transmission (24, 25), recent work has demonstrated consistently lower EIRs in rural compared to urban settings (3), suggesting that cities remain areas of comparatively low malaria transmission.

The African invasion by *An. stephensi* was first reported from Djibouti City in 2012 (26) and has since been recorded in Ethiopia (18, 27), Sudan (28, 29), Somalia, (30) and Somaliland (31), with recent work highlighting suitability of the continent’s largest population centers (where >100 million individuals live) as a habitat for this species (16). While causality has yet to be established, emergence of *An. stephensi* is thought to have contributed to resurgence of malaria transmission in Djibouti (10-fold increase in cases 2013 to 2019), highlighting the potential threat that this vector poses to malaria control across the Horn of Africa (32) and the continent more generally (33). Notably, *An. stephensi* has been identified across both rural and urban settings in the Horn of Africa to date (34, 35), highlighting the potential for this invasive vector to contribute to malaria transmission across a diverse range of different settings.

Despite the significant public health this vector poses, substantial uncertainty remains in how its establishment might influence malaria dynamics in the region, particularly in the (predominantly urban) settings where the disease is currently largely absent. A key driver of this will be the vector’s seasonal dynamics. Mosquito populations may show marked variation in seasonal abundance, often exhibiting substantial annual fluctuations in size that drive the temporal profile of disease risk. The efficacy of many malaria control interventions [such as seasonal malaria chemoprevention (36), indoor residual spraying (37) (IRS), or larval source management (38) (LSM)] depends on optimally timing their delivery relative to seasonal peaks in vector abundance. A better understanding of the seasonality of *An. stephensi* across its current range will help guide entomological monitoring and surveillance activities in areas of possible invasion and have material consequences for the effective control of *An. stephensi*-driven malaria transmission.

Here, we systematically collate longitudinal catch data for *An. stephensi* across its endemic range to better understand these dynamics. Our results highlight pronounced variation in the extent and timing of seasonality (poorly predicted by patterns of rainfall), with distinct dynamics separating rural and urban settings, with the latter tending to possess more seasonal dynamics on average. We show that this variation has material consequences for the effective design of entomological surveillance programs. Integrating these results with a previously published model of malaria transmission also highlights how this variation will influence the efficacy of malaria control efforts in parts of the Horn of Africa where the disease is currently (or has previously been) largely absent and underscores the need for rapid scale-up of entomological monitoring across the region.

## Methods

### Systematic Review of *An. stephensi* Surveys.

We collated references from published systematic reviews of literature relating to *An. stephensi* (16, 39) and updated these previous searches by searching Web of Science and PubMed from January 2017 to September 2020. We included all records containing temporally disaggregated adult mosquito catch data with monthly (or finer) temporal resolution spanning at least 10 mo that had not been conducted as part of vector control intervention trials and where at least 25 *An. stephensi* mosquitoes had been caught over the study period. A total of 36 references were collated containing 65 time series with monthly catch data (no study presented data at a finer temporal resolution) from surveys carried out across Afghanistan, Djibouti, India, Iran, Myanmar, and Pakistan. See *SI Appendix* for further details and references therein.

### Clustering of Similar Time Series and Random Forest Prediction of Cluster Membership.

Following methodologies developed in previous work (39), we fitted a gaussian process-based model to smooth these mosquito count time series using a negative binomial likelihood to account for overdispersion and a periodic kernel function to capture the repeating patterns often observed seasonally in mosquito populations. Model fitting was carried out within a bayesian framework using the probabilistic programming language Stan (40). We then calculated summary statistics for each smoothed time series to characterize their temporal properties (*SI Appendix*), generating a set of parameters for each time series that summarizes their temporal properties. We then scaled and normalized each summary statistic to give a mean 0 and unit variance—a process necessary for the PCA we apply to identify a lower-dimensional representation of the structure present in the data amenable to visualization.

Using k-means clustering, we identified clusters of time series with similar temporal properties—the output of this process is a label for each time series indicating which cluster (of time series with similar temporal properties) each specific time series was assigned to. For each study location, we extracted a suite of satellite-derived environmental variables (*SI Appendix*, Table S2) and used these variables alongside empirically calculated rainfall seasonality and average monthly catch as covariates within a random forest-based classification framework to predict cluster membership of each time series. These models were fitted using the R package *Ranger* (41) with sixfold cross-validation utilized to optimize hyperparameters. Results are based on averaging the results of 25 iterations of cross-validation and model fitting and predictions made using out-of-bag estimates. There were significant imbalances in class size across clusters and so we carried out upsampling using the Synthetic Minority Oversampling Technique (SMOTE) (42) algorithm. For results without upsampling, see *SI Appendix*.

### Probability of Detecting *An. stephensi* with Different Surveillance Strategies.

We explore the implications of seasonal variation in *An. stephensi* abundance on the probability of detecting the vector in entomological surveys using a theoretical sampling method with a defined amount of effort (such as a human landing catch conducted by a single volunteer for one night). We use a statistical framework (described further in *SI Appendix*) that calculates the cumulative probability of detection from i) an overall assumed *An. stephensi* annual biting rate (ABR, arbitrarily set to 20 for illustrative purposes here), ii) changes in vector density over the course of the year (from our collated time series), and iii) various factors relating to timing of, and effort expended in, the entomological survey. Specifically, for each time series, we identified the month with the highest rainfall and the month in which vector density was the highest (noting that these months were very rarely the same month). We then calculated the cumulative probability of *An. stephensi* detection using the framework under a range of different surveillance strategies. Specifically, three strategies were simulated as follows:

•  Vector Peak Timed: Starting the survey at the month with peak vector density (noting that in the absence of preexisting detailed entomological information, this is a hypothetical quantity designed to illustrate an approximate upper bound on the detection probability that could be achieved).
•  Rainfall Peak Timed: Starting the survey at the month with peak rainfall.
•  Random Month Timed: The expected probability of detection achieved if the survey was started during a random month (calculated by simulating survey starting in each of the year’s 12 mo and calculating the average cumulative probability).

In addition to varying the survey’s starting time, we also varied the amount of sampling effort (number of days sampled within each month) and overall duration of the survey (consecutive months sampled given a defined number of night sampling per month). Note that the aim here is not to describe the exact probability of missing *An. stephensi* in any given entomological survey as this will depend on a wide array of other, poorly defined and heterogeneous factors (e.g., type of catch methodology used and location). We also assume that the collection method is unbiased (i.e., not biased toward catching mosquitoes with particular resting or biting properties) which is also highly unlikely. Instead, the aim is to highlight how variation in seasonal dynamics can influence the nature of surveillance required to successfully detect a single *An. stephensi* (i.e., successfully establish presence), and the probability of detection should be viewed as a relative measure (i.e., viewed in relation to other sampling efforts and survey timings possible for surveys) and not an absolute value. Note that this framework assumes no seasonal variation in factors other than mosquito abundance (such as the ability of the sampling method to accurately record ABR) that might influence the probability of *An. stephensi* being caught by our theoretical sampling method.

### Modeling *An. stephensi*-Driven Malaria Dynamics and Control.

We integrated these vector abundance time series into a published population-level model of *P. falciparum* malaria transmission and disease dynamics (43–45) to explore the implications of *An. stephensi* seasonality on malaria control in settings in the Horn of Africa where malaria is currently largely absent (full description of the modeling framework is provided in *SI Appendix*). We use the modeling framework to understand how variation in mosquito seasonality might influence the impact of IRS, a key vector control intervention. As the dynamics of *An. stephensi*’s establishment and influence on temporal trends in malaria transmission during its establishment remain unclear, we focused on the time period immediately following establishment (when the disease is at equilibrium) and provide an illustrative example of how seasonality of *An. stephensi-*driven malaria transmission could influence the effectiveness of IRS in a site with no preexisting history of malaria control. For simplicity, we assume that all transmission is due to *An. stephensi* and that the IRS efficacy against this species is consistent with that observed against other species across the continent (46). We simulate the impact of a single, illustrative IRS campaign in a setting with an annual EIR of 1.5 (average malaria prevalence of 8 to 9%), timed for optimal impact, randomly or alongside peak rainfall, and assume that 80% of the vector’s resting sites are successfully sprayed (noting the vector is thought to also rest in animal houses which are not typically sprayed in public health campaigns). For further details, see *SI Appendix*.

## Results

### Diversity in Temporal Dynamics across the Collated *An. stephensi* Time Series.

**Fig. 1.**
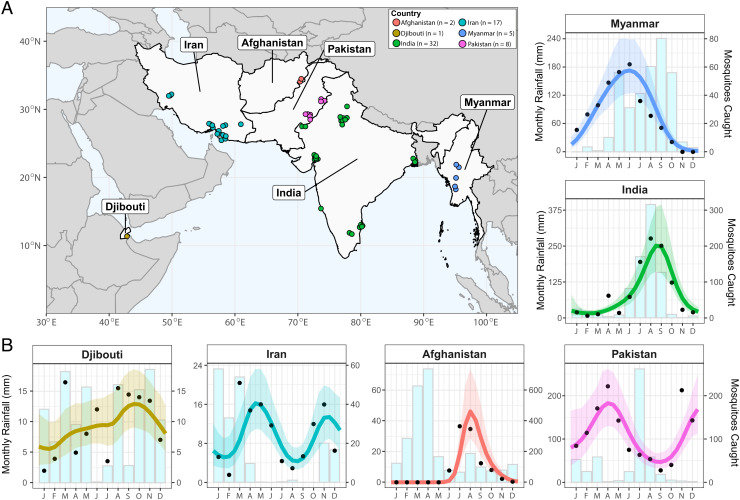
Sources and locations of *An. stephensi* time series data and examples for each country. (*A*) Map of the geographical range over which time series entomological collections have been carried out. Countries with studies are highlighted in light gray, and the locations of individual studies indicated by the individual points colored according to country (Afghanistan = red, Djibouti = yellow, India = green, Iran = turquoise, Myanmar = blue, and Pakistan = pink). (*B*) A single example *An. stephensi* time series from each country, with the empirical monthly mosquito catch (black points), fitted gaussian process curves (mean = colored line, ribbon = 95% bayesian credible interval) and monthly rainfall (matching sampling location and year of sampling) for each (light blue bars with gray surround). The *x*-axis indicates the month of sampling; the *y*-axis indicates either the monthly rainfall (left-hand side *y*-axis) or number of vectors caught in each month (right-hand side *y*-axis; note that the absolute number of mosquitoes caught between time series are not comparable due to variable sampling effort). n indicates the number of time series in each country.

A total of 65 time series from across Afghanistan, Djibouti, India, Iran, Myanmar, and Pakistan were identified (Fig. 1*A* and *SI Appendix*, Fig. S1). Substantial variation in the degree and timing of vector seasonality was observed, with the maximum percentage of annual vector density in any consecutive 4-mo period (a proxy for degree of seasonality) ranging from 35 to 99% across the collated studies (average = 62%). This contrasted with rainfall seasonality, where highly seasonal rainfall patterns were consistently observed across the locations the surveys had been carried out in (maximum percentage of annual rainfall in any consecutive 4-mo period, mean = 82 and range 47 to 99%). We also observed a diverse range of temporal patterns ranging from highly seasonal dynamics with a single seasonal peak to bimodal population dynamics with two peaks within a single year or more perennial patterns of abundance (Fig. 1*B*).

### Statistical Characterization and Clustering of Temporal Properties Highlight Distinct Archetypes.

**Fig. 2.**
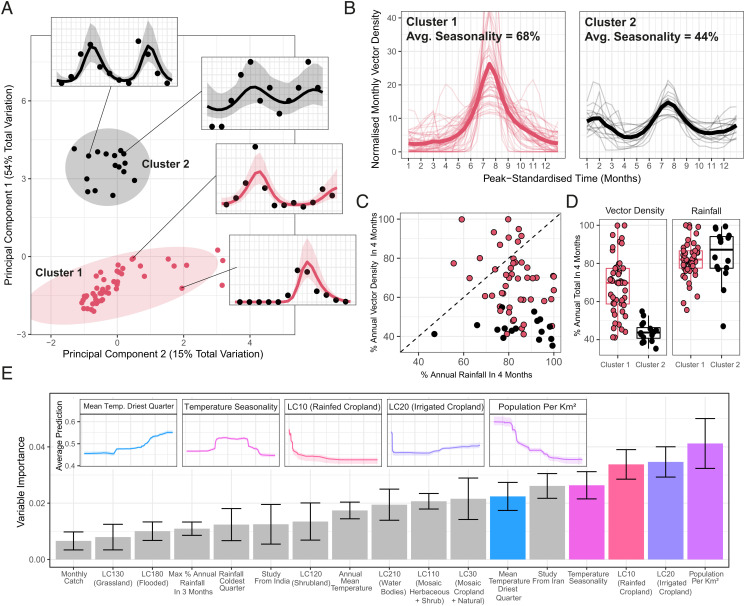
Characterization and clustering to identify time series with similar temporal properties. (*A*) Results of principal component analysis (PCA) and k-means clustering for two clusters. Points on the main figure indicate individual time series, with point color indicating cluster membership. Ellipsoids demarcate the 75th quantile of the density associated with each cluster. Principal components 1 and 2 are plotted together explaining 69% of the total variation in temporal properties across the time series. (*B*) Time series belonging to each cluster. Pale lines represent individual time series; brighter line indicates the mean of all the time series belonging to that cluster—in all cases, vector density is normalized to sum to 1 over the course of the year and time standardized so that the highest vector density for each time series is arbitrarily set to occur at month 7. (*C*) Plot comparing the percentage of annual total mosquito catch and percentage of annual total rainfall occurring in any consecutive 4-mo period for each time series colored by cluster membership. (*D*) Box plots show the percentage of annual total mosquito catch (*Left*) and annual total rainfall (*Right*) series occurring in any consecutive 4-mo periods for each time series. Rainfall data come from the *CHIRPS* dataset (37) and are specific to study location and time period. Each point indicates an individual time series. (*E*) Variable importance plot for each of the covariates included in the random forest model used to predict cluster membership—bar height indicates the mean variable importance across the 25 individual iterations of random forest fitting, with error bars representing the 95% CI. *Inset* plots are the partial dependence plots for the top five most important variables in the model showing how the average prediction for Cluster 2 (*y*-axis, with higher values indicating an increased probability of Cluster 2 membership) varies with (normalized) variable value (*x*-axis).

Summary statistics were calculated for each time series to characterize their temporal properties (*SI Appendix*, Fig. S2), followed by k-means clustering of the results to cluster the time series into groups with similar temporal patterns. Our results highlight two distinct clusters of time series (Fig. 2*A*), each characterized by distinct temporal patterns (Fig. 2*B*). Cluster 1 time series had single seasonal peaks and were more seasonal (average of 68% of annual vector density in the consecutive 4-mo period with the highest density) than Cluster 2 time series, which had more perennial patterns of annual abundance (average 44% of annual vector density in the consecutive 4-mo period with the highest density) and contained several time series with two peaks across the year. Despite differing significantly in mean vector abundance seasonality (Fig. 2 *C* and *D* and *P *< 0.001), there was no significant difference between clusters in rainfall seasonality (Fig. 2*D*, *P* = 0.59). Seasonality of rainfall (defined as the highest proportion of total annual rainfall occurring in any consecutive 4-mo period) across sampled locations was high (average 82% and 84% for Clusters 1 and 2, respectively) despite a wide variation in vector abundance seasonality. Timing of peak rainfall relative to peak vector density significantly differed between clusters (*SI Appendix*, Fig. S3), with peak rainfall and vector abundance separated by <1 mo on average for Cluster 1 compared to 2.2 mo for Cluster 2. There was, however, considerable within-cluster variation in timing—within Cluster 1, timing of peak vector density relative to rainfall ranged from −5.8 mo to +5.3 mo (with 6 mo the maximum gap that can occur within an annually repeating 12-mo time series, highlighting that the peaks in vector density relative to rainfall were found across the entirety of the year). We also explored varying the number of clusters specified in the k-means algorithm. Specifying 4 clusters resulted in further disaggregation of the 49 time series in Cluster 1 into 3 separate clusters, each characterized by a single seasonal peak, but which differed in the timing of peak vector density relative to peak rainfall (*SI Appendix*, Fig. S4).

### Random Forest Modeling of Seasonal Dynamics Highlights Urbanicity as a Key Factor.

We fitted a random forest-based classification framework to predict cluster membership (Cluster 1 or Cluster 2, as defined in Fig. 2*A*). Due to the significant class size imbalance between Cluster 1 (n = 49) and Cluster 2 (n = 16), we upsampled Cluster 2 data to generate balanced classes. Across 25 iterations of random forest model fitting, the mean area under the curve (AUC) was 0.89 (indicating good predictive performance), and the model was able to correctly classify Cluster 1 and Cluster 2 time series equally well (83% and 85% accuracy, respectively).

We calculated the relative importance of each variable to the model’s predictive ability (Fig. 2*E*). Patterns of land use were strongly associated with different clusters—time series from surveys in locations with lower population density (a proxy for rurality) more likely to belong to Cluster 2 (less seasonal) as were areas with a high proportion of land occupied by irrigated cropland. By contrast, a high proportion of land occupied by rainfed cropland was associated with Cluster 1 (more seasonal) dynamics. We also observed strong associations with temperature covariates, including the mean temperature of the driest quarter (where a high temperature was associated with Cluster 2), temperature seasonality (where a nonmonotonically increasing relationship was observed; for all covariate response plots, see Fig. 2 *E*,  *Inset* panels and *SI Appendix*, Fig. S5), and whether the study had been conducted in Iran (indicating potential spatial confounding). By contrast, rainfall seasonality was not an important predictor of temporal dynamics and was in the least five important variables. Examining the association between cluster membership and rurality/urbanicity (defined by the study authors), there was indication of an association (chi-squared test, *P* = 0 .07), although this was not statistically significant at the 5% level. About 88% (n = 22/25) of time series from urban settings were assigned to Cluster 1, and only 12% (n = 3/25) were assigned to Cluster 2. About 65% (n = 24/37) of time series from rural settings were assigned to Cluster 1 and 35% (n = 13/37) to Cluster 2. We also explored how seasonality varied with both cluster membership and rurality/urbanicity (*SI Appendix*, Fig. S6). There was indication that rural time series assigned to Cluster 1 (mean seasonality 71%) were on average more seasonal than urban time series assigned to Cluster 1 (mean seasonality 64%), although this result was not statistically significant at the 5% level (*P *= 0.06, *t* test). No difference in seasonality between Cluster 2 time series carried out in rural (average seasonality 44%) and urban (average seasonality 45%) settings was observed (*P *= 0.59, *t* test).

Model predictive performance and variable importance rankings were similar when no upsampling was applied (AUC = 0.81, *SI Appendix*, Fig. S7), although predictive accuracy was highly unbalanced (Cluster 2 accuracy = 50% and Cluster 1 accuracy = 94%). Model performance and variable importance ordering remained similar when fitting the model and explicitly holding out a subset of the data to subsequently evaluate model performance (n = 7 time series, *SI Appendix*, Fig. S8). Predictive power for seasonality (percentage of vector catch in any 4-mo period) was more modest, although estimates were positively correlated (r = 0.43, *SI Appendix*, Fig. S9).

### Implications of Seasonal Dynamics for Entomological Surveillance of *An. stephensi* across the Horn of Africa.

**Fig. 3.**
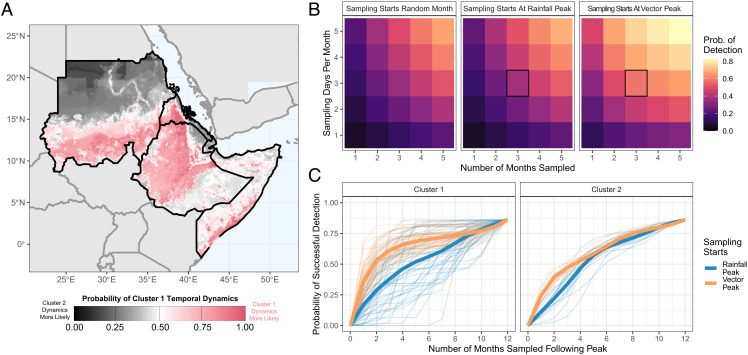
Possible seasonal dynamics of *An. stephensi* across the Horn of Africa, and consequences for entomological surveillance and monitoring. (*A*) Environmental covariates were collated across countries in the Horn of Africa where *An. stephensi* has been found, and the random forest classification model from Fig. 2*E* used to predict potential temporal dynamics. Map shows the probability of temporal dynamics belonging to Cluster 1 (more seasonal), with pink corresponding to Cluster 1 dynamics being more likely, and black indicating Cluster 2 dynamics (more perennial) are more likely, with white indicating both are equally likely. (*B*) For a setting with an ABR of 20, the average probability (across all 65 collated *An. stephensi* temporal profiles) of detecting *An. stephensi* (where detection is defined as catching ≥1 mosquito) for a range of different sampling efforts (number of consecutive months sampled and number of sampling days in each month) in a setting with an ABR of 20 bites per person. These results were generated for three different sampling strategies: i) with sampling starting at a random month in the year (*Left*-hand panel, subsequently averaged over all possible sampling start months in the year), ii) with sampling starting in the month of peak rainfall (*Center* panel), and iii) with sampling starting in the month of peak vector density (*Right*-hand panel). (*C*) For setting with an ABR of 20 and a sampling effort of 3 d per month, the cumulative probability of *An. stephensi* detection as a function of the number of consecutive months sampled for each individual time series stratified by sampling strategy (starting at peak vector abundance = orange and at peak rainfall = blue) and cluster. In both panels, pale thin lines indicate the cumulative probability curve for a specific temporal profile, and thicker lines indicate the average for the specific sampling strategy.

We collated the same covariates for countries across the Horn of Africa and used the random forest model to predict cluster membership and potential temporal dynamics of *An. stephensi* across the region (Fig. 3*A*). Our results highlight distinct geographical areas considered more likely to belong to Cluster 1 (more seasonal) and Cluster 2 (less seasonal) and areas of significant uncertainty. We next asked what consequences this seasonality might have on entomological surveillance of the vector, with a focus on how these seasonal dynamics might interact with features of surveillance programs such as the timing and duration of entomological surveys. Across the collated temporal profiles, in a setting with an ABR of 20, surveys consisting of 3-mo sampling and three sampling days per month that were timed to start at periods of peak *An. stephensi* density were on average 64% more likely to detect the vector compared to starting the survey at a random month of the year and 57% more likely to successfully detect the vector compared to starting the survey in the month of peak rainfall (Fig. 3*B*). Timing of entomological surveys to coincide with peaks in rainfall did not lead to a significant increase in the probability of successfully detecting *An. stephensi* (average 4% increase), suggesting that the timing of peak rainfall may be a poor measure for guiding entomological surveys searching for the vector. We next stratified these results by temporal cluster (Fig. 3*C*). For Cluster 1 (and a survey lasting 3 mo, with a 3-d sampling per month), we observed differences in the cumulative probability of detection when comparing strategies which start surveys at the location’s rainfall peak compared to starting them at peak *An. stephensi* abundance—on average, the latter strategy increased the cumulative probability of detection by 62% compared to a randomly timed survey compared to only a 40% increase over random timing for Cluster 2.

### Modeling the Impact of *An. stephensi* Seasonality on Vector Control Measures.

**Fig. 4.**
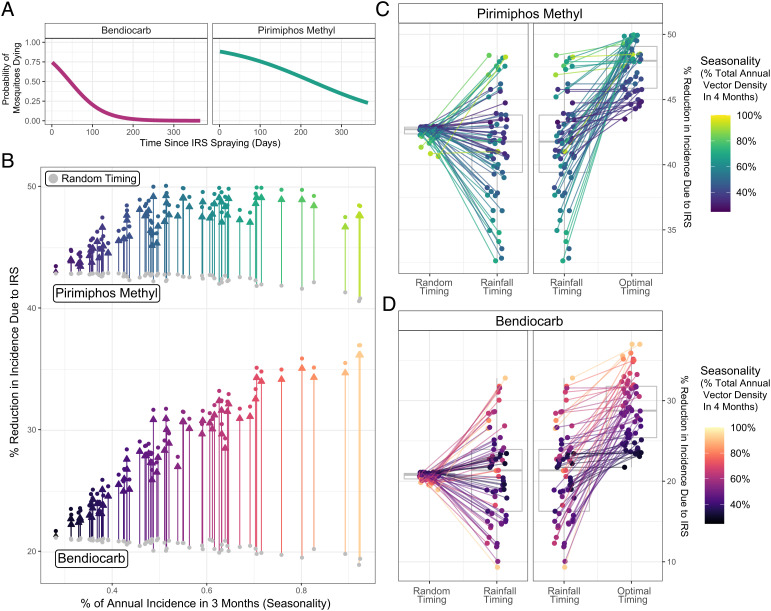
Modeling the public health impact of IRS and the influence of *An. stephensi* seasonality. (*A*) Probability of mosquitoes dying upon exposure to each IRS compound in the time period following spraying (46)—pink indicates bendiocarb, and turquoise indicates pirimiphos-methyl. (*B*) For each temporal profile, the public health impact of annual IRS campaign with each insecticide according to the timing of the campaign. Points in gray correspond to the average reduction in incidence occurring from picking a random month to conducting the IRS campaign for each *An. stephensi* temporal profile; colored points indicate the reduction in incidence arising from optimally timing the IRS campaign relative to the vector density peak for each *An. stephensi* temporal profile (and colored arrows indicate the difference). The arrows and points are colored according to i) the insecticide used and ii) the degree of *An. stephensi* seasonality in each temporal profile (defined as the proportion of total annual abundance in any consecutive 4-mo period). (*C*) For pirimiphos-methyl and for each *An. stephensi* temporal profile (colored points), the percentage reduction in malaria incidence achieved if the IRS campaign is timed randomly, timed to start when rainfall is at its peak, or optimally timed based on peak vector density. Points correspond to specific *An. stephensi* temporal profiles and are colored according to their degree of seasonality. A box plot shows the minimum, first quartile, third quartile, and maximum for the different individual projections. (*D*) As for (*C*) but for the insecticide bendiocarb.

Integrating the temporal profiles of *An. stephensi* abundance with a malaria transmission model, we explored how variation in temporal dynamics influences the impact of IRS (with two different insecticides, Fig. 4*A*). Across the *An. stephensi* temporal profiles, optimal timing of IRS delivery resulted in an average of 47.6% reduction in annual malaria incidence in the 12 mo following spraying for pirimiphos-methyl and 28.9% for bendiocarb (Fig. 4*B*). These results represent 1.12× and 1.41× increases over the average impact achieved if the campaign is timed to a random month of the year. The extent to which optimal timing provided greater impact than random timing was dependent on the degree of seasonality and insecticide, however—it increased with the degree of seasonality and was consistently larger for bendiocarb than pirimiphos-methyl (due to the latter’s longer duration and retention of residual activity following spraying). Timing of the IRS campaign to occur when rainfall peaks did not significantly increase impact compared to timing of the campaign to a random month [with less than a 2% average increase in impact for both pirimiphos-methyl (Fig. 4*C*) and bendiocarb (Fig. 4*D*)] had significantly lower impact than optimally timed campaigns (39% and 15% lower impact for pirimiphos-methyl and bendiocarb, respectively).

## Discussion

Invasion and establishment of *An. stephensi* across the Horn of Africa represents an urgent threat to malaria control in the region. Understanding the temporal profile of vector abundance of the species will inform effective deployment of surveillance, monitoring, and control interventions aimed at mitigating this potential impact, particularly in urban settings where malaria has historically been largely absent or only minimally present. Collating data from across the vector’s endemic range, we identify broad diversity in the extent and nature of *An. stephensi* seasonal dynamics. This variation is associated with a wide array of ecological factors, including seasonal fluctuations in temperature and patterns of land use, including a potential role of urbanicity in shaping dynamics.

Our analyses identified population per km^2^ as the most important predictor of cluster membership, with high population density (a proxy for urbanicity) being strongly associated with Cluster 1 dynamics (more seasonal patterns of abundance). Intriguingly, while urban settings were frequently seasonal, our analyses highlighted a wide diversity of dynamics in rural settings—ranging from seasonal peaking dynamics (that were, on average, more seasonal than urban settings) to bimodal or more perennial patterns of abundance (that were less seasonal than the dynamics observed in urban settings). This potential disparity in temporal dynamics across rural and urban settings will likely have implications for both how resources aimed at surveillance control should be targeted to these different settings and the public health impact of different control interventions.

It is important to note however that the statistical associations identified here do not represent causal statements, and so further fieldwork exploring *An. stephensi*’s niche (both across its historical range and the Horn of Africa) is needed to identify the mechanisms underpinning the different dynamics. A related limitation is the absence of sufficiently spatially granular data to explore seasonal patterns at subcity resolution and assess potential differences in dynamics across typically heterogeneous urban landscapes. These caveats notwithstanding, our results suggest that urban *An. stephensi* populations are likely to display more seasonal dynamics, supporting the utility of temporally targeted interventions like short-lived IRS or LSM in these settings. The same is not necessarily true in rural settings, where shorter-duration control interventions are likely to be impactful but may be less consistent in their effectiveness (without local surveys being conducted to establish the timing and extent of seasonality) due to the range of seasonal profiles observed, which included more perennial patterns of abundance. This is before considering other factors that differ between urban and rural settings [e.g., predominant household building material and wall structure (47)] that may contribute to differences in the efficacy between rural and urban settings and that are not considered here. Furthermore, implementing these measures and achieving sufficient intervention population coverage in urban settings is likely to present logistical challenges, given the historical absence of large-scale vector control campaigns from urban communities. If these barriers can be surmounted, however, our results suggest such measures are likely to be impactful, although remaining uncertainty around the degree of endophily *An. stephensi* can display (48) might necessitate alternative interventions to IRS that are not dependent on resting behavior, such as LSM (38).

Our results also suggest a limited role for rainfall in shaping the diverse temporal dynamics across the collated *An. stephensi* catch data contrary to results observed for other African malaria vector species [e.g., *An. Gambiae* (49, 50)]. Specifically, that areas with highly seasonal rainfall may not have highly seasonal patterns of *An. stephensi* abundance. Instead, our analyses highlight an association between temperature and seasonal patterns of abundance with both temperature seasonality and the average temperature during the driest quarter being highly predictive of dynamics. This is consistent with previous work identifying temperature as a key driver of mosquito population dynamics due to its impact on an array of mosquito life history traits including biting rate, life span, and fecundity (among several others) (51, 52).

We also observed a strong association with land use, with cropland being associated with different dynamics depending on whether it was irrigated or rainfed. The absence of a relationship linking seasonal dynamics and rainfall seasonality when considering our collated data in aggregate might therefore arise from the diverse array of land use contexts in which surveys were carried and how they interact with incipient rainfall, as well as the interactions of other factors such as temperature, which may limit mosquito breeding and survival even when water is available for breeding. Rainfall is clearly important—but its impact on mosquito abundance is likely mediated by the structure of the local environment (including both land use and climactic factors such as temperature). It should be stressed however that these covariates identified here are not necessarily predictive of absolute *An. stephensi* abundance in a region but rather the seasonality in abundance. A much more detailed sampling strategy considering variability in the accuracy and biases of sampling methods and other geospatial methods will be needed to identify whether the vector has invaded a region.

The work also highlights the exceptionally limited amount of longitudinally collected entomological data from across *An. stephensi's* current geographical range (including the Horn of Africa region) that currently exists. In highly seasonal settings, there is a risk of erroneously concluding *An.stephensi*’s absence, particularly as the time of low vector catches may not coincide with times of low rainfall, as is frequently the case for other mosquitoes endemic to Africa. Longitudinal surveys enabling better description of these dynamics would therefore be useful in enabling subsequent refinement and timing of shorter surveys aimed at detecting presence only (while also providing additional information on temporal dynamics that can facilitate the effective targeting and timing of interventions such as IRS or LSM). Indeed, our results suggest that rainfall may provide a poor guide to timing of intervention campaigns in settings where *An. stephensi* is the dominant vector, underscoring the crucial role detailed entomological data collection and establishment of patterns empirically will play in optimizing vector surveillance and disease control efforts.

There are several important limitations to the work presented here. First, we assume that the inferred ecological relationships linking environmental features to temporal dynamics will translate from the vector’s historical range to the Horn of Africa. Indeed, our results highlight significant plasticity and variation in *An. stephensi*’s seasonal abundance depending on the setting, and therefore, the extent to which our results will extrapolate to new settings remains unclear—making collection and analysis of longitudinal catch data collected from the Horn of Africa an urgent research priority. Relatedly, due to the limited amount of data available and the wide geographical range over which the collated studies were conducted, we cannot rule out the possibility of spatial confounding in shaping the inferred associations. Analysis of the distribution of locations stratified by rural/urban status and cluster assignment did not reveal obvious patterns of spatial confounding (*SI Appendix*, Fig. S10), although the study being conducted from Iran was a high-ranking variable in the random forest model, and it is possible that some degree of spatial confounding is present. Additionally, the limited spatial resolution typically available for our collated data precluded finer-scale evaluation of seasonal dynamics and meant our analyses were limited to using coarse environmental covariates that are likely imperfect proxies for the underlying drivers of dynamics (e.g., population per km^2^ as a proxy for urbanicity and recognizing that the urban landscape itself is typically highly heterogeneous across a single city). Moreover, the use of population per km^2^ as a proxy for urbanicity neglects the likely significant variation between urban locations in ecological factors relevant to anopheline seasonal dynamics (such as patterns of water storage or severity of pollution); factors we were unable to explore in the results presented here.

We were also unable to consider the possibility of variation in temporal dynamics between *An. stephensi* forms. Identification of the *An. stephensi* form is challenging, requiring close visual examination (53) or molecular methods (54). Availability of these data was limited, and we lack the ability to disaggregate time series by the specific form caught and hence preclude form as a confounder of some of the identified relationships linking environmental factors and temporal dynamics. Another limitation relates to our usage of mosquito abundance data, which is highly prone to biases driven by the collection method used. Collections were carried out using different sampling methods, locations, and timings, although there was insufficient information in most studies to include these covariates within the analyses. Biases associated with the trapping method, location, and/or timing might vary between seasons, such as the comparative probability of detection indoors vs. outdoors [and its interaction with the vector’s well-documented resistance to insecticides (55)], or vary depending on the time of day when sampling was occurring. These biases could affect the reliability of the seasonal patterns inferred here and the probability of detecting *An. stephensi* under different sampling efforts.

We do not include insecticide resistance into our model of malaria transmission. Extensive insecticide resistance has been demonstrated for *An. Stephensi* (55–57), and recent populations assayed in Ethiopia showed resistance to all four major insecticide classes (34, 58), suggesting that IRS might have a lower impact than suggested here. Relatedly, we do not consider uncertainty in *An. stephensi* bionomic properties (e.g., timing of biting or whether resting occurs predominantly indoors or outdoors), which might vary by season and could further modulate the impact of interventions such as IRS where killing is mediated primarily through indoor resting following feeding. Variation in *An. stephensi*’s bionomic properties has previously been identified (59), including a propensity for crepuscular biting and resting outside of houses compared to other *Anopheles* species dominant in sub-Saharan Africa (16, 19, 48) that might render IRS less effective and necessitate consideration of other strategies such as LSM. While the aim of this work is to illustrate how seasonality modulates intervention impact, these considerations underscore the urgent need for a more detailed characterization of *An. stephensi* across the Horn of Africa to quantify its bionomic properties and insecticide resistance profile, as well as more detailed quantification of larval dynamics and breeding site utilization, which will be required for effective LSM deployment. Indeed, these will be crucial inputs to future work evaluating the range of potential interventions (including but not limited to IRS, as is the case with the work presented here) aimed at controlling *An. stephensi* to identify the most effective package to deploy.

Our work highlights significant variation in temporal dynamics across *An. stephensi* populations; variation that is shaped by distinct ecological factors can markedly differ between urban and rural settings, which has material consequences for the effectiveness of vector control interventions. Our work also highlights the need to better understand the vector’s dynamics in settings where it has newly established, how these dynamics might differ from and interact with other *Anopheles* species also present, and the mechanistic relationships underpinning these different responses to factors such as urbanization. Indeed, the trajectory of *An. stephensi*’s establishment and subsequent dynamics in the Horn of Africa remains deeply unclear, and the scarcity of published entomological studies from the region underscores the need for studies longitudinally surveying locations where *An. stephensi* has recently arrived. This will be important to understanding the patterns of seasonal variation the vector displays and support optimizing the delivery of malaria control interventions aiming to mitigate the impact of this invasive vector.

## Supplementary Material

Appendix 01 (PDF)

Dataset S01 (CSV)

## Data Availability

All data collated as part of this study and the code required to reproduce these analyses can be found at the following link: https://github.com/cwhittaker1000/stephenseasonality (60).
